# Quality of life in patients with chronic hand eczema

**DOI:** 10.1192/j.eurpsy.2022.2296

**Published:** 2022-09-01

**Authors:** M. Bouhamed, R. Sallemi, K. Sallemi, I. Feki, J. Masmoudi

**Affiliations:** 1 Hospital university of HEDI CHAKER, Psychiatry A Department, Sfax, Tunisia; 2 hedi chaker hospital, Dermatology, sfax, Tunisia

**Keywords:** chronic hand eczema, Quality of Life

## Abstract

**Introduction:**

Chronic hand eczema (CHE) , inflammatory dermatitis, can lead to physical and psychosocial disability altering the quality of life of these patients.

**Objectives:**

The objective of this study was to examine the quality of life in patients with chronic hand eczema

**Methods:**

Descriptive study collating patients who consulted for CHE, at the Dermatology Department of the CHU Hédi Chaker Sfax, during 3 years (2018-2020). A socio-demographic, clinic , and the Quality of life Questionnaire (DLQI) were administered in this study.

**Results:**

Our study included 12 patients (8 men and 4 women). The mean age was 46.8±11.6 years. The patients were in professional activity in 86.8% of the cases. No patient was in early retirement or disability status. The average duration of the disease was 4.5 years (1- 9 years). All patients were in remission. The intensity of pruritus at the last attack was mild (25.77%), moderate (72.23%), and severe (2%). The impact of pruritus on sleep was noted in 100%. The mean total quality of life score (DLQI) was 6.8±5.5 which means a moderate impairment of quality of life

**Conclusions:**

This work highlights the importance of the impact of this dermatitis on the quality of life of these patients. Therefore, multidisciplinary dermatological and psychiatric management is considered necessary

**Disclosure:**

No significant relationships.

